# Probiotic and functional potential of lactic acid bacteria isolated from pulque and evaluation of their safety for food applications

**DOI:** 10.3389/fmicb.2023.1241581

**Published:** 2023-09-12

**Authors:** Yesica Ruiz-Ramírez, Rogelio Valadez-Blanco, Concepción Calderón-García, Michael Leonidas Chikindas, Edith Ponce-Alquicira

**Affiliations:** ^1^Departamento de Biotecnología, Universidad Autónoma Metropolitana Unidad Iztapalapa, Iztapalapa, Mexico; ^2^Instituto de Agroindustrias, Universidad Tecnológica de la Mixteca, Oaxaca, Mexico; ^3^Health Promoting Naturals Laboratory, School of Environmental and Biological Sciences, Rutgers, The State University of New Jersey, New Brunswick, NJ, United States; ^4^Center for Agrobiotechnology, Don State Technical University, Rostov-on-Don, Russia; ^5^Department of General Hygiene, I.M. Sechenov First Moscow State Medical University, Moscow, Russia

**Keywords:** lactic acid bacteria, probiotics, antioxidant activity, β-galactosidase activity, cholesterol reduction, bile salts hydrolase, virulence factor, antibiotic resistance

## Abstract

Pulque is a traditional Mexican non-distilled alcoholic beverage to which several beneficial functions are attributed, mainly associated with gastrointestinal health, which can be explained by the presence of probiotic bacteria in its microbiota. Therefore, the objective of this work was to evaluate the safety, probiotic activity, and functional characteristics of seven strains of lactic acid bacteria (LAB) isolated from pulque using the probiotic strain *Lactobacillus acidophilus* NCFM as control. The LAB isolates were identified by 16S rRNA sequencing and MALDI Biotyper^®^ MS as belonging to three different *Lactobacillaceae* genera and species: *Lactiplantibacillus plantarum*, *Levilactobacillus brevis* and *Lacticaseibacillus paracasei*. Most strains showed resistance to gastric juice, intestinal juice and lysozyme (10 mg/L). In addition, all strains exhibited bile salt hydrolase (BSH) activity and antibacterial activity against the pathogenic strain *Listeria monocytogenes*. Additionally, cell surface characteristics of LAB were evaluated, with most strains showing good hydrophobicity, auto-aggregation, and co-aggregation towards enteropathogenic *Escherichia coli* and *L. monocytogenes*. In terms of safety, most of the strains were sensitive to the tested antibiotics and only the *Lact. paracasei* UTMB4 strain amplified a gene related to antibiotic resistance (*mecA*). The strains *Lact. plantarum* RVG2 and *Lact. plantarum* UTMB1 presented γ-hemolytic activity, and the presence of the virulence-related gene *agg* was identified only in UTMB1 strain. Regarding functional characterization, the tested bacteria showed good β-galactosidase activity, antioxidant activity and cholesterol reduction Based on principal component analysis (PCA) and heat mapping, and considering the strain *Lact. acidophilus* NCFM as the probiotic reference, the strains *Lacticaseibacillus paracasei* UTMB4, *Lactiplantibacillus plantarum* RVG4 and *Levilactobacillus brevis* UTMB2 were selected as the most promising probiotic strains. The results of this study highlighted the probiotic, functional and safety traits of LAB strains isolated from pulque thus supporting the health benefits attributed to this ancestral beverage.

## 1. Introduction

Probiotics are defined as “live microorganisms that, when administered in adequate amounts, confer a health benefit on the host,” among which one of the most important is the regulation of the gastrointestinal microbiota (Hill et al., 2014). In this regard, it is well documented the ability of probiotics to relieve or prevent some human diseases based on different physiological mechanisms such as lactose tolerance and immune-system enhancement, antimicrobial and anticarcinogenic activities, among others (Markowiak and Ślizewska, 2017; Kerry et al., 2018).

A large number of lactic acid bacteria (LAB) have been employed for the manufacture of a wide variety of fermented foods and beverages, based on their ability to improve their preservation, sensory, nutritional and functional attributes (Zannini et al., 2016; Vijayalakshmi et al., 2020). Currently, LAB are largely recognized for their ability to produce several antimicrobial substances, such as weak organic acids, hydrogen peroxide, bacteriocins and others (Rastogi et al., 2022).

Recently, it has been of great interest the ability of some LAB, such as *Lacticaseibacillus paracasei*, to reduce the cholesterol blood levels in people with hypercholesterolemia and their action for preventing atherosclerosis (Chaiyasut et al., 2021). The mechanisms for the alleviation of hypercholesterolemia by probiotics may be associated with the antioxidant activity that induces anti-inflammatory activity and modulates cholesterol metabolism. Considering that hypercholesterolemia is a major risk factor associated with cardiovascular diseases, and one of the leading causes of death worldwide, the discovery of new cholesterol-lowering drugs is of paramount importance in current medicine (Khongrum et al., 2023).

An important feature in the evaluation of new probiotics is safety properties such as antibiotic resistance and virulence factors. At present, it is known that safety properties are highly strain-specific: therefore, each strain must be thoroughly evaluated (Pradhan et al., 2020; Lakhlifi et al., 2023). Antibiotic resistance is considered a global health problem and has been rapidly increasing, the mechanism mainly associated with this problem is the transfer of genes from one microorganism to another; therefore, probiotics should not carry transferrable antibiotic resistance markers (Pradhan et al., 2020; Fan et al., 2023). Hemolytic activity is a common virulence factor in pathogenic microorganisms that facilitates the acquisition of iron and causes anemia in the host (Fan et al., 2023). Other virulence factors influence adherence to host tissue, invasion, abscess formation, resistance and modulation of host defense mechanisms, secretion of cytolysins and production of plasmid-encoded pheromones (Alvarez-Cisneros et al., 2017). *In vitro* and *in silico* methods are the most rapid methods for safety assessment and would be the most suitable before moving on to *in vivo* evaluations.

Pulque is an ancestral Mexican beverage obtained from the fermentation of the sap extracted from various species of *Agave* (aguamiel). It is characterized by being a non-distilled, white, viscous beverage with low alcoholic content (Escalante et al., 2016; Gutiérrez-Uribe et al., 2017). In the 1930s the production of pulque suffered a dramatic collapse which was associated with the social and economic instability during the Mexican Revolution war and the growing demand for other alcoholic beverages, such as beer, which was aggravated by a strong discrediting campaign of the techniques used for pulque production. Currently, this product has regained scientific and public interest, not only because of the findings of its nutritional and functional benefits but also as a source of complex microbiota associated with its fermentation (Escalante et al., 2016).

Numerous studies on the pulque microbiota have highlighted the remarkable abundance of LAB during the fermentation stage and in the final product (Escalante et al., 2016). These bacteria are responsible for the lactic acid fermentation in pulque and contribute importantly to the sensory characteristics of the beverage. In addition, several reports have demonstrated that pulque microorganisms are responsible for a number of benefits traditionally associated with this product, particularly those related to the prevention or amelioration of gastrointestinal diseases (Chacón-Vargas et al., 2020). In this regard, many *in vitro* studies have been conducted demonstrating the outstanding probiotic potential of LAB from pulque, particularly from strains belonging to the *Leuconostoc* genus and the *Lactobacillaceae* family (Castro-Rodríguez et al., 2015; Giles-Gómez et al., 2016). The probiotic activity tests carried out on pulque LAB strains *in vitro* include resistance to gastrointestinal tract conditions (acid pH, bile salts and lysozyme), inhibitory activity against foodborne pathogens, hydrophobicity, antibiotic resistance and hemolytic activity (Castro-Rodríguez et al., 2015; Giles-Gómez et al., 2016; Torres-Maravilla et al., 2016). In addition, Castro-Rodríguez et al. (2015) evaluated the adhesion of 4 strains of *Leuconostoc mesenteroides* isolated from aguamiel to the intestinal wall of 3-month-old male Wistar rats, reporting adhesion values from 79 to 83%. However, to date, there are no reports in the literature regarding the health benefits of pulque strains.

In previous work, we evaluated the probiotic potential of eleven LAB isolated from pulque obtained from different locations in the states of Oaxaca and Puebla, using the widely characterized probiotic strain *Lactobacillus acidophilus* NCFM as a positive control (Ruíz-Ramírez et al., 2022). The strains were identified by 16S rRNA sequencing as *Lactiplantibacillus plantarum*, *Levilactobacillus brevis, Lacticaseibacillus paracasei* and *Liquorilactobacillus ghanensis*. The tested strains were characterized by a noticeable resistance to pH 2.0, 0.5% bile salts, and antimicrobial activity against *Listeria monocytogenes, Escherichia coli* (EPEC) and *Salmonella enterica* serovar Typhi. Considering the above, the objective of this work was to carry out a comprehensive study of the probiotic, functional and safety traits of LAB strains isolated from pulque, thus contributing to the revalorization of this ancestral Mexican beverage.

## 2. Materials and methods

### 2.1. Biological material

In this work, we studied seven LAB strains previously evaluated for their probiotic potential and antimicrobial activity (Ruíz-Ramírez et al., 2022), isolated from pulque. The probiotic strain *Lactobacillus acidophilus* NCFM, kindly provided by Danisco USA, was used as a positive control in all the tests performed in this work. The following microorganisms were used as controls in the probiotic potential and safety tests: enteropathogenic *Escherichia coli* (EPEC) 2348/69, *Salmonella enterica* serovar Typhi ATCC 9992 and *Enterococcus faecalis* ATCC 29212 from the Culture Collection of the Faculty of Chemistry, Universidad Nacional Autónoma de México; *Pediococcus acidilactici* MXVK133 (GenBank: JQ783346), *Enterococcus faecium* SF68, *Listeria monocytogenes* LM-W207 (GenBank: OQ733281) and *Staphylococcus aureus* SA-W207 (GenBank: OQ733284) from the Culture Collection of the Laboratory of Molecular Biology and Biochemistry of Macromolecules, Universidad Autónoma Metropolitana-Unidad Iztapalapa, México.

### 2.2. Bacterial identification by 16S rRNA gene sequencing and MALDI Biotyper^®^ mass spectrometry

The identification of the strains was conducted firstly by sequencing the 16S rRNA gene of the bacterial samples. DNA extraction was performed using the Ultra Clean^™^ – Microbial DNA isolation kit (Mo Bio Laboratories Inc., Carlsbad, CA, USA). The integrity of the DNA was verified on a 1% agarose gel and visualized on the gel documentation system Biotop Fluor Shot EVO (BIOTOP, Huangshan City, China). Subsequently, the 16S rRNA gene was amplified from the DNA obtained using the solution Taq PCR Master Mix (QIAGEN GmbH, Hilden, Germany) and the universal primers 27F and 1492R (Angmo et al., 2016). The conditions used for the PCR reaction were as follows: denaturation (94°C, 3 min), amplification (94°C, 45 s; 52°C, 30 s; 72°C, 1 min × 30 cycles) and a final amplification at 72°C for 10 min. Finally, the amplification products were purified with the GeneJet PCR purification kit (Thermo Fisher Scientific Baltics, Vilnius, Lithuania) and sequenced by Macrogen Inc. (Seoul, Republic of Korea), the MEGA 11 software was used to build the phylogenetic tree using the Neighbor- Joining method (Ruíz-Ramírez et al., 2022).

As a supporting tool for species-level identification, MALDI Biotyper^®^ mass spectrometry was employed at the Divisional Mass Spectrometry Laboratory of the Universidad Autónoma Metropolitana (Rodrigues et al., 2021). Individual isolated colonies were picked from MRS agar plates and placed into 1.5 mL vials, after which, ethanol, formic acid, and acetonitrile were added sequentially for the inactivation and rupture of the cell membrane and its precipitation by centrifugation. Subsequently, 1 μL of the supernatant was placed on a steel plate (Bruker, Karlsruhe, Germany) with 1 μL of the matrix α-ciano-4-hydroxycinnamic acid (Sigma-Aldrich, Burlington, MA, USA). The analysis was performed with a MALDI Biotyper^®^ MS Autoflex speed mass spectrometer (Bruker Daltonics, Bremen, Germany) using the MALDI Biotyper^®^ tool. The resulting data were compared to the MBT Compass database to assign the genera and the species of the LAB strains.

### 2.3. Resistance to simulated gastrointestinal conditions

In order to test the ability of the LAB microorganisms to survive their transit to the gastrointestinal tract, studied strains were subjected *in vitro* to the following simulated gastrointestinal conditions: exposure to lysozyme, gastric juice (GJ), intestinal juice (IJ) and GJ/IJ. In addition, in this study it was assessed the antimicrobial and bile salt hydrolase activities of the LAB strains.

#### 2.3.1. Resistance to lysozyme

The resistance to lysozyme (Sigma-Aldrich) was determined according to Torres-Maravilla et al. (2016), with some modifications. LAB strains were inoculated in MRS broth, followed by incubation at 37°C for 20 h under microaerophilic conditions. Subsequently, 2 mL of the culture was centrifuged at 4500 × *g* for 6 min at 4°C, after which the pellet was subjected to two washes with PBS. Following this, the culture was adjusted to a DO_600_ of 1.0 using a Ringer solution. Finally, 10% of the cell culture was suspended in a sterile electrolyte solution containing 10 mg/L of lysozyme, after which the suspension was incubated at 37°C for 3 h. The resistance of the test strains to the presence of lysozyme was determined by the spread-plate count method in duplicate (Ruíz-Ramírez et al., 2022).

#### 2.3.2. Resistance to gastric juice (GJ) and intestinal juice (IJ)

The *in vitro* resistance of LAB strains to gastric (GJ) and intestinal (IJ) juices was performed separately and sequentially. Fresh colonies of the test LAB strains were inoculated in 10 mL of MRS broth, and incubated at 37°C for 20 h under microaerophilic conditions. Following this, the bacterial culture was centrifuged at 4500 × *g* for 6 min at 4°C, after which the pellet was subjected to two washes with PBS and adjusted to 10^8^–10^9^ CFU/mL. Subsequently, an aliquot of the cell culture was separately inoculated (10%) in gastric juice (PBS with 0.3% pepsin and adjusted to pH 2.0 with HCl) (Kumari et al., 2022; Zhang et al., 2022) and in intestinal juice (PBS with 0.3% bile salts, 0.1% pancreatin and adjusted to pH 8.0 with NaOH) (Zhou et al., 2022) and incubated at 37°C for 3 h under microaerophilic conditions. Finally, in the combined treatment, the GJ culture was inoculated at 10% in the IJ (GJ/IJ) and incubated at 37°C for 3 h in microaerophilic conditions (Zhang et al., 2022). As a control for the assay, the cell culture was inoculated in PBS at pH 7. The logarithmic percentage of resistance to JG, JI and JG/JI was determined (Ruíz-Ramírez et al., 2022).

#### 2.3.3. Bile salts hydrolase (BSH) activity

The BSH activity of the tested LAB strains was determined by the agar diffusion method. LAB-impregnated filter paper discs (6 mm) were superimposed on MRS agar plates supplemented with 0.5% (w/v) bile salts and CaCl_2_ (0.375 g/L). Following this, the discs were incubated under microaerophilic conditions at 37°C for 48 h (Zheng et al., 2013). The BHS activity was evaluated by measuring the diameter of the halo formed around the disc with a digital caliper.

#### 2.3.4. Antibacterial activity

The antibacterial activity of the tested strains was evaluated by the agar diffusion method (da Silva Ferrari et al., 2016). Prior to the test, crude extracts (CE) were obtained from 20-h LAB cultures by centrifugation at 6500 × *g* for 15 min under refrigeration conditions (4°C). Subsequently, the supernatant was recovered, and the pH was adjusted to 7.0 to eliminate the influence of organic acids. Following this, the extract was filtered through 0.22 μm pore-size sterile membranes and stored at −80°C. In addition, CE-impregnated filter paper discs (6 mm) were superimposed on TBS agar previously inoculated with 140 μL of *L. monocytogenes* LM-W207 suspension (10^6^–10^7^ CFU/mL). Two positive controls were used (15 μL) with nisin and lysozyme at 10% concentration. The antibacterial activity was evaluated by measuring with a digital caliper the inhibition zone diameter of the halo formed around the disc after the plate was incubated at 37°C for 24 h.

### 2.4. Cell-surface properties

The cell surface characteristics evaluated in this work were auto-aggregation, co-aggregation and hydrophobicity, since these surface properties have been associated with the ability of bacteria to adhere to the intestinal epithelium (Castro-Rodríguez et al., 2015; Śliżewska et al., 2021).

#### 2.4.1. Hydrophobicity

The hydrophobicity test was carried out by the method of microbial adhesion to solvents (MATS) (Castro-Rodríguez et al., 2015; Kang et al., 2018). LAB cultures in their exponential phase were pelleted by centrifugation at 5000 × *g* for 30 min, followed by two washes and resuspension in PBS (pH 7.2) to get an OD_600_ of 1. Following this, the suspension was separately mixed with different organic solvents (chloroform and hexane) in a 1:1 ratio, followed by vortex stirring for 30 s. The mixture was incubated for 1 h at room temperature, after which the absorbance of the aqueous phase was measured at 600 nm. The hydrophobicity (H) was calculated with the following equation:

$$H\left[\%\right]=\left(\frac{A_{0}-A}{A_{0}}\right)*100$$

where A_0_ and A indicate the absorbance before and after extraction with organic solvents, respectively.

#### 2.4.2. Auto-aggregation

The auto-aggregation assay was performed using exponential-phase LAB cultures. After incubation, the cell cultures were centrifuged at 5000 × *g* for 10 min at 4°C. Subsequently, the cell pellet was washed twice with PBS pH 7.2, after which the pellet was resuspended in PBS to an OD_600_ of 0.5. The suspension was incubated at 25°C and auto-aggregation was monitored by measuring the absorbance (600 nm) at different times: 0, 2, 4, 6, 20, and 24 h (Śliżewska et al., 2021). Auto-aggregation (A) was calculated as:

$$A\left[\%\right]=\left(\frac{A_{0}-A}{A_{0}}\right)*100$$

where A_0_ is the initial absorbance and A the absorbance at different times.

#### 2.4.3. Co-aggregation

For the co-aggregation assay, bacterial suspensions were prepared as described in the auto-aggregation test (Śliżewska et al., 2021). The LAB strains were mixed separately with *L. monocytogenes* LM-W207*, Salmonella enterica* serovar Typhi ATCC 9992 and EPEC in equal volumes and incubated at 25°C (Zuo et al., 2015). The test was monitored by measuring the OD_600_ at different times: 2, 4, 6, 20 and 24 h. The extent of co-aggregation (C) was calculated as:

$$C\left[\%\right]=\frac{\left[\left(A_{x}+A_{\mathrm{LAB}}\right)-\left(A_{\mathrm{mix}}\right)\right]}{\left[\left(A_{x}+A_{\mathrm{LAB}}\right)\right]}*100$$

where A_x_ is the initial absorbance of *L. monocytogenes* LM-W207*, S. enterica* serovar Typhi ATCC 9992 or EPEC; A_LAB_ is the initial absorbance of the LAB strain; and A_mix_ is the absorbance of the mixture at the different testing times.

### 2.5. Safety traits

The safety traits tested in this work involved the antibiotic resistance against common antibiotics, hemolytic activity and study of the expression of genes associated with virulence factors.

#### 2.5.1. Antibiotic resistance

For testing the antibiotic resistance of the studied strains, the disk diffusion method was employed (Sakandar et al., 2018; Colombo et al., 2020). LAB colonies (4 to 5) were transferred from a 36-h MRS cultured plate to a tube containing 4–5 mL of physiological serum, followed by incubation at 37°C until reaching a turbidity comparable to the 0.5 McFarland standard. After adjusting the turbidity of the suspension, a sterile swab was dipped into it and streaked thoroughly on Mueller Hinton agar plates. The plates were left to dry for 3–5 min, after which antibiotic discs (6 mm) were placed on the surface of the agar. The antibiotic discs Combi Disc Multidisc for Gram Positive GP1 (Accutrack, Mexico City, Mexico) as well as vancomycin (30 μg) (PiSA, Mexico City, Mexico) were used for testing a total of 15 antibiotic compounds. Subsequently, the plates were incubated at 37°C for 18–24 h, after which each plate was examined by measuring the diameters of inhibition zones. The results are reported as sensitive (≥30 mm), intermediate (16–29 mm) and resistant (≤15 mm), according to the inhibition zones obtained for the different antibiotics (Sakandar et al., 2018). EPEC was used as the control of the test.

A PCR assay was performed to determine the presence of antibiotic resistance genes in the test strains from pulque and in the probiotic control (*Lact. acidophilus* NCFM) (Boyle-Vavra and Daum, 2010; Alvarez-Cisneros et al., 2017). Genomic DNA extraction was performed using the Ultra CleanTM – Microbial DNA isolation kit (MO BIO Laboratories Inc., San Diego, CA, USA) and verified on a 1% agarose gel. PCR reactions were performed according the manufacturer using the Phusion^™^ High-Fidelity DNA polymerase (Thermo Fisher Scientific Baltics, Vilnius, Lithuania) with the addition of each primer at 10 μM (Table 1) and genomic DNA at 0.5 μg/μL. The PCR reaction was performed in a Mastercycler EP Gradient thermocycler (Eppendorf, Hamburg, Germany) programmed for each gene with the following conditions: *vanA* (95°C, 7 min; 35 × [95°C, 30 s; 58°C, 30 s; 72°C, 2 min]; 72°C, 10 min); *mecA* (95°C, 7 min; 35 × [95°C, 30 s; 55°C, 30 s; 72°C, 2 min]; 72°C, 10 min); *tetM* (94°C, 5 min; 35 × [94°C, 30 s; 55°C, 30 s; 72°C, 2 min]; 72°C, 10 min); and *tetS* (94°C, 5 min; 35 × [94°C, 30 s; 50°C, 30 s; 72°C, 2 min]; 72°C, 10 min). As positive controls, the following genes were used: *Pediococcus acidilactici* MXVK133 (*vanA*), *Staphylococcus aureus* SA-W207 (*mecA, tetM* and *tetS*), *Listeria monocytogenes* LM-W207 (*mecA*) and *Enterococcus faecalis* ATCC 29212 (*tetM*) and EPEC (*mecA*). The PCR products were verified on a 1.5% agarose gel using a 100 bp DNA ladder (Invitrogen, Carlsbad, CA, USA).

**Table 1:** Primers used for the detection of virulence factors and antibiotic resistance (Boyle-Vavra and Daum, 2010; Alvarez-Cisneros et al., 2017).

Gene	Description	Primer (sequence 5′-3′)	Product size (pb)
*mecA*	Penicillins	CTTTGCTAGAGTAGCACTCG GCTAGCCATTCCTTTATCTTG	500
*tetM*	Tetracyclines (factor M)	ACAGAAAGCTTATTATATAAC TGGCGTGTCTATGATGTTCAC	171
*tetS*	Tetracyclines (factor S)	GAAAGCTTACTATACAGTAGC AGGAGTATCTACAATATTTAC	169
*vanA*	Vancomycin type A	GGCTGCGATATTCAAAGCTC CCGGCTTAACAAAAACAGGA	157
*cyIM*	Postranslational modification of cytolysin	CTGATGGAAAGAAGATAGTAT TGAGTTGGTCTGATTACATTT	742
*esp*	Enterococcal surface protein	TTGCTAATGCTAGTCCACGACC GCGTCAACACTTGCATTGCCGAA	933
*cpd*	Pheromone cPD1 lipoprotein	TGGTGGGTTATTTTTCAATTC TACGGCTCTGGCTTACTA	782
*ace*	Collagen adhesin	AAAGTAGAATTAGATCCACAC TCTATCACATTCGGTTGCG	350
*geIE*	Gelatinase	ACCCCGTATCATTGGTTT ACGCATTGCTTTTCCATC	419
*cyIA*	Activation of cytolysin	TGGATGATAGTGATAGGAAGT TCTACAGTAAATCTTTCGTCA	517
*ccf*	Pheromone cCF10 precursor lipoprotein	GGGAATTGAGTAGTGAAGAAG AGCCGCTAAAATCGGTAAAAT	543
*agg*	Collagen binding cell wall	AAGAAAAAGAAGTAGACCAAC AAACGGCAAGACAAGTAAATA	1,553
*efaA_fs_*	*Enterococcus faecalis* specific cell-wall adhesion	GACAGACCCTCACGAATA AGTTCATCATGCTGTAGTA	705
*efaA_fm_*	*Enterococcus faecium* specific cell-wall adhesion	AACAGATCCGCATGAATA CATTTCATCATCTGATAGTA	735

#### 2.5.2. Detection of virulence factors

To determine the presence of genes associated with virulence factors in the test LAB strains and in the control, PCR experiments were performed (Alvarez-Cisneros et al., 2017). The reactions were performed according to Phusion^™^ High-Fidelity DNA Polymerase (Thermo Fisher Scientific Baltics), with the addition of each primer at 20 μM (Table 1) and 0.5 μg/μL genomic DNA. The PCR reaction was performed in a Mastercycler EP Gradient thermocycler (Eppendorf) programmed with the following conditions: 94°C, 2 min; 30 × [94°C, 1 min; 54°C, 1 min; 72°C, 1 min]; 72°C, 5 min. The following genes were used as positive controls: EPEC (*efaA*_fs_), *Enterococcus faecium* MXVK29 (*efa*A_fm_) and *Enterococcus faecalis* MXVK133 for the rest of the genes. The PCR products were verified on a 1% agarose gel using a 1 kb DNA ladder (Thermo Fisher Scientific Baltics).

#### 2.5.3. Hemolytic activity

The hemolytic activity was determined by streaking a colony of the tested LAB strains or the control on Columbia agar medium added with 5% defibrinated blood (Castro-Rodríguez et al., 2015). The inoculated plates were incubated at 37°C for 24 h. The appearance and color of the halos are indicative of the type of hemolytic activity: (i) transparent, β-hemolytic activity; (ii) green, α-hemolytic; (iii) no halo, γ-hemolytic (Castro-Rodríguez et al., 2015; Touret et al., 2018). *L. monocytogenes* LM-W207 was used as the positive control.

### 2.6. Functional traits

The evaluation of the potential physiological functionality of the tested LAB strains was carried out by determining *in vitro* the antioxidant and β-galactosidase activities, as well as their capacity for cholesterol reduction. These functional traits are some of the most widely reported in the literature for highly efficient probiotic strains.

#### 2.6.1. Antioxidant activity

The antioxidant activity was determined according to Rezaei et al. (2020) and Kaur et al. (2017), with some modifications. Colonies of the test strains with 36 h growth were inoculated in MRS broth and incubated for 20 h at 37°C. The cell pellet was recovered by centrifugation (6000 × *g*, 10 min at 4°C) followed by two washes with double distilled water. Subsequently, the pellet was resuspended and adjusted to 10^10^ UFC/mL based on the McFarland standard. Following this, the cell suspension was mixed with a 0.004% (w/v) DPPH ethanolic solution in a 1:1 ratio. The mixture was agitated vigorously and incubated at room temperature in darkness for 30 min. As a blank of the test, the reaction mixture above was prepared using double distilled water instead of the cell suspension and its absorbance was measured at 517 nm (A_blank_). Finally, the reaction mixture was centrifuged at 8000 × *g* for 10 min and the absorbance of the supernatant was measured at 517 nm (A_sample_). The percentage of antioxidant activity (AA) was calculated with the following equation:

$$\%\mathrm{AA}=100*\left[1-\left(\frac{A_{\mathrm{sample}}}{A_{\mathrm{blank}}}\right)\right]$$

#### 2.6.2. β-galactosidase activity

To assess β-galactosidase activity, the LAB strains were streaked in lactose medium plates (0.5% lactose, 0.5% peptone, 0.3% meat extract and 1.5% bacteriological agar) added with 50 μg/mL of the chromogenic compound 5-bromo-4-choro-3-idolyl-β-galactopyranoside (X-gal; Sigma-Aldrich). The plates were incubated at 37°C for 24–48 h. The presence of blue-stained colonies was indicative of β-galactosidase activity (Gheytanchi et al., 2010; Kaur et al., 2017).

#### 2.6.3. Cholesterol reduction

For the cholesterol reduction assay, the LAB strains were cultured in MRS broth and incubated at 37°C for 20 h. Additionally, an MRS broth added with 0.5% of bile salts and 100 μg/mL of cholesterol previously dissolved in ethanol was prepared. The tested strains were inoculated in the MRS-cholesterol broth (1% inoculum concentration) followed by incubation for 24 h at 37°C (Das et al., 2016). An MRS-cholesterol broth added with 1% MRS broth without inoculation was used as the blank. Subsequently, the suspensions were centrifuged at 3000 × *g* for 10 min, the supernatants were recovered, and the absorbance was determined at 570 nm. The percentage of cholesterol reduction (CR) was calculated with the following equation:

$$CR\;\left[\%\right]=\frac{A_{b}-A_{\mathrm{LAB}}}{A_{b}}x100$$

where A_blank_ and A_LAB_ are the absorbance of the blank and of the suspension with the LAB bacteria, respectively.

### 2.7. Statistical and multivariate analysis

One-way ANOVA analyses were performed at a 95% significance level to evaluate whether there was a significant difference between the different conditions evaluated. In addition, for mean comparison analyses, Tukey’s tests were performed considering a significance level of 95% (*p* < 0.05). Minitab 17 software (Minitab Inc., USA) was used. Principal component analysis (PCA), heat map and scatter plot were performed to visualize and study multivariate data clustering in RStudio v.1.4.1106, using the plot, biplot and heatmap functions, respectively.

## 3. Results

### 3.1. Bacterial identification

**Figure 1:**
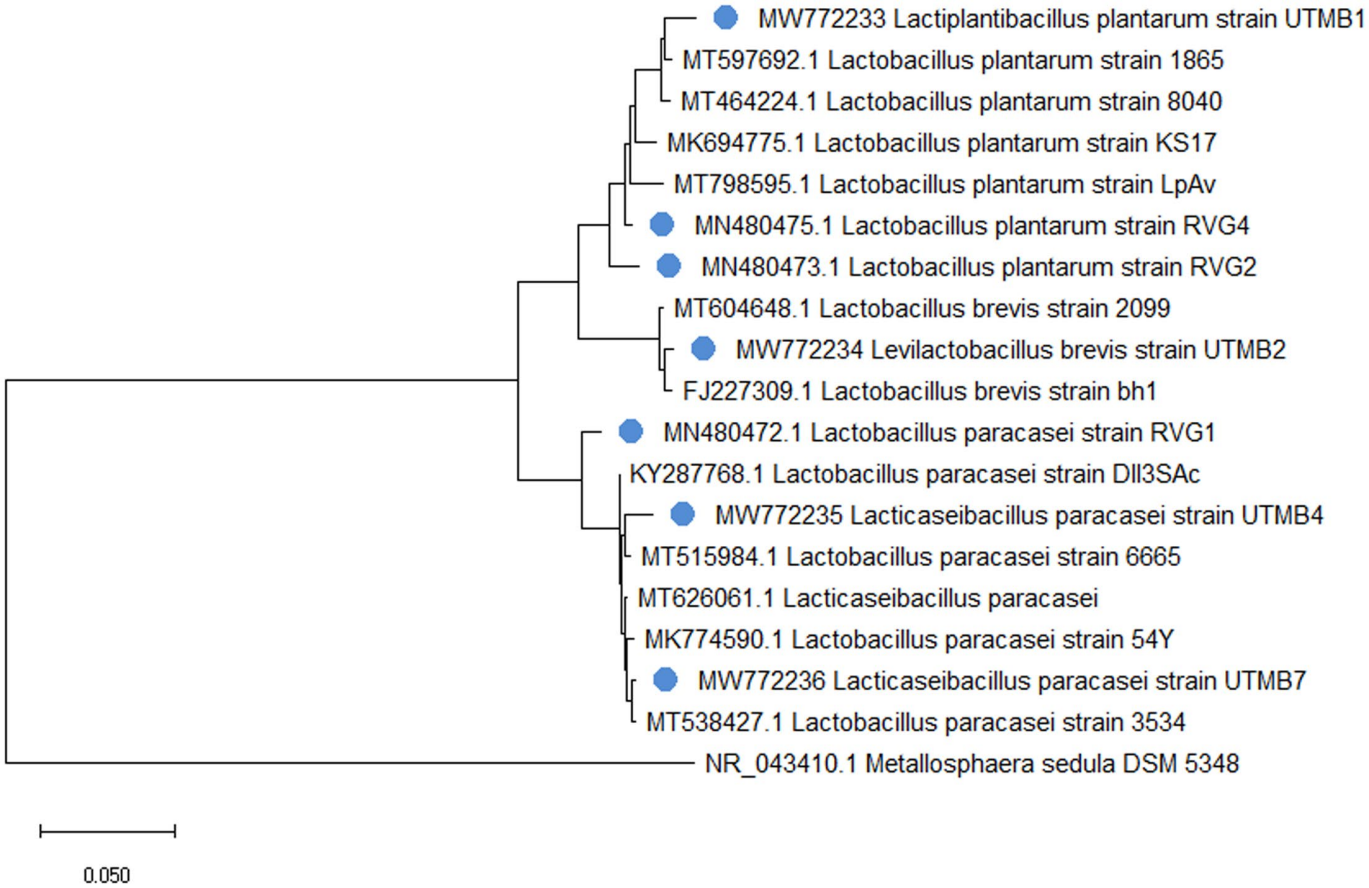
Phylogenetic tree of identified 16S rRNA sequences from pulque LAB and reference sequences obtained from the GenBank database. The pulque’s BALs are marked with a blue circle and each one has its own accession numbers. *Metallosphaera sedula* ARS120-2 was used as an outgroup reference strain.

The identification of pulque LAB strains was conducted by sequencing of the 16S rRNA gene and corroborated by MALDI Biotyper® mass spectrometry. It was determined that the studied strains belong to the *Lactobacillaceae* family presenting 97–99% homology with sequences of microorganisms deposited in the GenBank database of the NCBI, corresponding to the following species: *Lacticaseibacillus paracasei, Levilactobacillus brevis,* y *Lactiplantibacillus plantarum* (Table 2). Figure 1 shows the phylogenetic tree of pulque LAB. The identification of the LAB strains was corroborated by MALDI Biotyper^®^ mass spectrometry. Typing results of the bacterial strains by MALDI Biotyper^®^ software are shown in Table 2, with identification scores ranging between 2.10 and 2.45.

**Table 2:** Results of the identification of the lactic acid bacteria strains from pulque by 16S rRNA gene sequencing and MALDI Byotiper^®^ mass spectrometry.

Strain	Taxon	Gene bank access number	BLAST NCBI	MALDI Biotyper^®^ MS
			% Identity	Score
RVG1	*Lacticaseibacillus paracasei*	MN480472.1	97.02	2.20
RVG2	*Lactiplantibacillus plantarum*	MN480473.1	99.05	2.26
RVG4	*Lactiplantibacillus plantarum*	MN480475.1	97.9	2.47
UTMB1	*Lactiplantibacillus plantarum*	MW772233	98.64	2.44
UTMB2	*Levilactobacillus brevis*	MW772234	98.84	2.29
UTMB4	*Lacticaseibacillus paracasei*	MW772235	98.43	2.46
UTMB7	*Lacticaseibacillus paracasei*	MW772236	99.72	2.46

### 3.2. Resistance to gastrointestinal conditions

#### 3.2.1. Simulated gastrointestinal tract conditions

After exposing the LAB strains to lysozyme, the strains presented survival percentages between 51 and 91% (Table 3), which were in all cases greater than that of the control *Lact. acidophilus* NCFM (47.5%). The LAB strains studied in this work presented survival to gastric juice ranging from 26 to 49.3%, whereas the commercial probiotic strain *Lact. acidophilus* NCFM had a survival rate of 56% (Table 3). In addition, the resistance of the bacterial strains to intestinal juice was assessed, resulting in survival rates between 91 and 99%, which are comparable to that obtained with the strain *Lact. acidophilus* NCFM (93.9%, Table 3). Finally, in the test for simulating the overall gastrointestinal transit (GJ/IJ), the LAB strains studied in this work presented a survival rate ranging from 25 to 44%, whereas the commercial probiotic strain *Lact. acidophilus* NCFM had a survival rate of 40% (Table 3).

**Table 3:** *In vitro* characterization of the probiotic potential of pulque lactic acid bacteria.

Strain	Logarithmic survival rate (3 h)				Antibacterial activity (*L. monocytogenes*, mm)^a^	BSH activity (0.5% BS, mm)
	GJ	IJ	GJ/IJ	Lysozyme		
*Lact. paracasei* RVG1	26.9 ± 0.1 ^e^	94.12 ± 0.5 ^d^	25.0 ± 1.2 ^f^	91.0 ± 1.4 ^a^	9.8 ± 0.8 ^a, b, c^	15.5 ± 1.3 ^d^
*Lact. plantarum* RVG2	48.4 ± 0.2 ^b^	91.1 ± 0.0 ^e^	39.0 ± 0.2 ^b, c^	90.9 ± 0.1 ^a^	9.7 ± 0.6 ^b, c^	20.7 ± 2.1 ^b, c^
*Lact. plantarum* RVG4	43.4 ± 0.6 ^c^	99.4 ± 0.3 ^a, b^	44.2 ± 0.4 ^a^	83.4 ± 0.5 ^c^	7.5 ± 0.5 ^d^	21.1 ± 1.0 ^b, c^
*Lact. plantarum* UTMB1	36.8 ± 0.6 ^d^	98.8 ± 0.0 ^b^	29.1 ± 0.3 ^e^	88.1 ± 0.8 ^b^	9.2 ± 0.8 ^b, c, d^	22.0 ± 1.0 ^b^
*Lact. brevis* UTMB2	36.7 ± 0.2 ^d^	99.5 ± 0.4 ^a, b^	31.2 ± 0.0 ^d^	73.9 ± 0.4 ^e^	9.5 ± 0.5 ^b, c^	27.7 ± 1.2 ^a^
*Lact. paracasei* UTMB4	26.1 ± 0.2 ^e^	97.3 ± 0.1 ^c^	30.1 ± 0.4 ^d, e^	82.5 ± 0.2 ^c^	8.2 ± 0.3 ^c, d^	18.7 ± 0.6 ^c, d^
*Lact. paracasei* UTMB7	49.3 ± 0.1 ^b^	99.9 ± 0.0 ^a^	38.2 ± 0.2 ^c^	65.8 ± 0.6 ^f^	10.0 ± 1.0 ^a, b^	17.0 ± 1.0 ^d^
LA^b^	56.1 ± 0.3 ^a^	93.9 ± 0.2 ^d^	40.3 ± 1.1 ^b^	47.5 ± 0.4 ^g^	11.5 ± 0.5 ^a, c^	28.7 ± 0.6 ^a^

Resistance to gastrointestinal tract barriers: high acidity (pH = 2), bile salts (0.3% BS) and presence of lysozyme; and antibacterial activity against *Listeria monocytogenes;* and bile salts hydrolase (BSH) activity (0.5% BS). Results are expressed as the mean ± SD (*n* = 2). The letters correspond to the Tukey test results (*p* < 0.05), different letters in each column indicate significant difference in the Tukey test. nd, not determined.

^a^ Lysozyme 12.0 ± 1.0 mm; Nisin 18.3 ± 1.5 mm.

^b^ LA, *Lactobacillus acidophilus* NCFM control strain.

#### 3.2.2. Bile salts hydrolase (BSH) activity

The BSH activity of the LAB strains was assessed. All tested strains showed BSH activity, including the control, presenting inhibition zones ranging from 15.5 to 28.7 mm (Table 3). A higher lysis of bile-salts was observed for the strains *Lactiplantibacillus plantarum* RVG2, *Lactiplantibacillus plantarum* RVG4, *Lactiplantibacillus plantarum* UTMB1 and *Levilactobacillus brevis* UTMB2.

#### 3.2.3. Antibacterial activity

In the antibacterial activity assay, the LAB strains of the study presented inhibition zones ranging from 6.9 to 9.1 mm (Table 3). *Lacticaseibacillus paracasei* UTMB7 presented the largest inhibition zone (9.1 mm) among the tested strains, corresponding statistically to the same level of antibacterial activity as the control *Lact. acidophilus* NCFM (8.8 mm). The antibacterial activities of the positive controls lysozyme and nisin were 13.4 mm and 11.3 mm, respectively.

### 3.3. Cell–surface properties

The hydrophobicities of the LAB strains were assessed using two organic solvents: chloroform (Chl) and hexane (Hex) (Table 4), with hydrophobicity values lying between 16 and 91%, and from 2.9 to 53%, respectively. *Levilactobacillus brevis* UTMB2 was the strain that presented the highest hydrophobicities among the tested bacteria (Chl: 91.5%, Hex: 53.0%), with values comparable to those presented by the control strain *Lact. acidophilus* NCFM (Chl: 95.7%, Hex: 55.2%), but statistically different (*p* < 0.05).

**Table 4:** Characteristics of the cell surface of pulque lactic acid bacteria: hydrophobicity (chloroform and hexane), auto-aggregation and co-aggregation (EPEC, *Listeria monocytogenes* and *S. typhi*).

Strain	%Hydrophobicity		%Auto-aggregation^b^	%Co-aggregation^b^		
	Chloroform	Hexane		EPEC	*L. monocytogenes*	*S. typhi*
*Lact. paracasei* RVG1	22.0 ± 0.2 ^g^	6.1 ± 0.3 ^h^	17.0 ± 0.3 ^g^	58.6 ± 0.1 ^f^	83.7 ± 0.5 ^d^	87.3 ± 0.4 ^a, b^
*Lact. plantarum* RVG2	29.4 ± 0.3 ^e^	2.9 ± 0.3 ^i^	27.0 ± 0.1 ^c^	64.9 ± 0.1 ^a^	85.8 ± 0.2 ^b, c^	83.6 ± 0.2 ^f^
*Lact. plantarum* RVG4	36.1 ± 0.3 ^d^	19.7 ± 0.3 ^d^	21.9 ± 0.2 ^e^	61.4 ± 0.1 ^d^	85.3 ± 0.3 ^c^	86.5 ± 0.2 ^b, c^
*Lact. plantarum* UTMB1	16.6 ± 0.1 ^h^	8.1 ± 0.2 ^g^	15.5 ± 0.3 ^h^	62.3 ± 0.1 ^c^	83.6 ± 0.1 ^d^	83.4 ± 0.1 ^f^
*Lact. brevis* UTMB2	91.5 ± 0.4 ^b^	53.0 ± 0.3 ^b^	34.3 ± 0.5 ^b^	59.5 ± 0.1 ^e^	83.8 ± 0.3 ^d^	85.4 ± 0.1 ^d, e^
*Lact. paracasei* UTMB4	26.9 ± 0.2 ^f^	12.6 ± 0.3 ^e^	20.3 ± 0.1 ^f^	64.4 ± 0.1 ^b^	86.6 ± 0.0 ^a, b^	85.9 ± 0.2 ^c, d^
*Lact. paracasei* UTMB7	25.8 ± 1.0 ^f^	21.6 ± 0.3 ^c^	19.5 ± 0.0 ^f^	62.2 ± 0.1 ^c^	84.7 ± 0.0 ^c, d^	84.7 ± 0.3 ^e^
LA^a^	95.7 ± 0.1 ^a^	55.2 ± 0.3 ^a^	94.1 ± 0.1 ^a^	64.1 ± 0.0 ^b^	87.6 ± 0.4 ^a^	88.2 ± 0.4 ^a^

Results are expressed as the mean ± SD (*n* = 2). The letters correspond to the Tukey test results (*p* < 0.05), different letters in each column indicate significant difference in the Tukey test.

^a^ LA, *Lactobacillus acidophilus* NCFM control strain.

^b^ 24 h incubation.

The percentages of auto-aggregation and co-aggregation were evaluated at 2, 4, 6, 20, and 24 h of incubation (Supplementary materials 1, 2, respectively). The auto-aggregation capacity of the tested bacteria at 24 h of incubation ranged from 15 to 34% (Table 4) and the strain *Levilactobacillus brevis* UTMB2 (34.3%) presented the highest capacity among the pulque strains. On the other hand, the auto-aggregation value for the control strain, *Lact. acidophilus* was far more superior (94%) than that of the tested LAB strains.

The results of the co-aggregation capacity of the LAB strains with EPEC, *L. monocytogenes* and *S. typhi*, are shown in Table 4. The tested strains showed a high co-aggregation capacity with all co-aggregate strains. The co-aggregation capacities at 24 h of the LAB strains lied in the following ranges: 83.4 to 88.2%, 83.6 to 87.6%, and 58.5 to 64.9%, with the co-aggregates *S. typhi*, *L. monocytogenes* and EPEC, respectively. In general, higher co-aggregation percentages were observed with *L. monocytogenes* and *S. typhi* co-cultures. The control *Lact. acidophilus* NCFM presented co-aggregation values of 88.2, 87.6, and 64%, with *S. typhi*, *L. monocytogenes* and EPEC, respectively. *Lactiplantibacillus plantarum* RVG2 presented the highest co-aggregation value for EPEC, which was even greater than that for the control strain. *Lacticaseibacillus paracasei* UTMB4 and *Lacticaseibacillus paracasei* RVG1 presented the highest co-aggregation values for *L. monocytogenes* and *S. typhi*, which were statistically similar to that of the probiotic control.

### 3.4. Safety assessment

The results of the antibiotic resistance of the tested strains are presented in Table 5. The LAB test strains mostly presented sensitivity and intermediate sensitivity to the antibiotics used in the agar diffusion assay. In addition, in the PCR assay, most of the tested strains did not present antibiotic resistance genes, except for *Lact. paracasei* UTMB4 which showed the presence of the gene associated with beta-lactam resistance (*mecA*) (Table 6). The *mecA, tetM, tetS* and *vanA* genes were successfully amplified in their respective positive controls. On the other hand, concerning the genes associated with virulence factors, only the gene *agg* was amplified in the *Lact. plantarum* UTMB1 strain (Table 6). The virulence-related genes tested in this work were successfully amplified in their respective positive controls. In addition, the tested strains were evaluated for their hemolytic activity in a blood culture medium (Table 5). No halos were observed around the bacterial streaks of most of the tested strains including the negative control *Lact. acidophilus* NCFM indicating a ɣ-hemolytic behavior. Only the strains *Lact. plantarum* UTMB1 and *Lact. plantarum* RVG2 presented α-hemolytic activity, whereas *L. monocytogenes* LM-W207, the positive control, presented β-hemolytic activity.

**Table 5:** Antibiotic susceptibility for the disk diffusion method and hemolytic activity of pulque lactic acid bacteria.

Strain	Antibiotic susceptibility^a^															Hemolytic activity^b, c^
	AMX	AMC	AZ	CFM	CFZ	XM	CI	C	SXT	EM	OF	P	PI	TE	VAN	
RVG1	I	S	S	R	I	I	R	I	I	S	I	I	S	S	I	ɣ
RVG2	S	S	S	S	I	S	R	S	I	S	R	S	S	I	S	α
RVG4	I	S	I	I	I	I	R	I	I	I	R	I	I	I	S	ɣ
UTMB1	S	S	I	I	I	I	R	I	S	I	R	I	I	I	I	α
UTMB2	I	S	I	I	I	I	R	I	I	I	R	I	I	I	S	ɣ
UTMB4	S	S	S	R	I	I	I	I	I	S	I	I	S	S	S	ɣ
UTMB7	S	S	I	R	R	I	I	S	I	I	R	I	S	S	I	ɣ
LA^d^	S	S	S	I	I	S	I	S	I	S	S	S	S	I	S	ɣ
EPEC ^e^	I	I	R	I	I	I	I	I	S	R	I	R	I	S	I	nd

Antibiotic codes: AMX = amoxicillin; AMC = amoxicillin/clavulanic acid; AZ = azithromycin; CFM = cephalexin; CFZ = cefazolin; XM = cefuroxima; CI = ciprofloxacin; C = chloramphenicol; SXT = cotrimoxazole; EM = erythromycin; OF = ofloxacin; P = penicillin; PI = piperacillin; TE = tetracycline; VAN = vancomycin.

nd, not determined.

^a^ Antibiotic susceptibility codes: S = Sensitive ((≥30 mm); I = Intermediate (16–29 mm); R = Resistant (≤15).

^b^ Transparent halo, β-hemolytic activity; (ii) green halo, α-hemolytic; (iii) no halo, ɣ-hemolytic.

^c^ β-hemolytic activity with *L. monocytogenes* (positive control).

^d^ LA, *Lactobacillus acidophilus* NCFM control strain.

^e^ EPEC, enteropathogenic *Escherichia coli*.

**Table 6:** Safety aspects of pulque lactic acid bacteria: genes associated with antibiotic susceptibility and virulence factors genes.

Strain	Antibiotic resistance				Virulence factors									
	*mecA*	*tetM*	*tetS*	*vanA*	*cyIM*	*esp*	*cpd*	*ace*	*geIE*	*cyIA*	*ccf*	*agg*	*efaA_fs_*	*efaA_fm_*
RVG1	−	−	−	−	−	−	−	−	−	−	−	−	−	−
RVG2	−	−	−	−	−	−	−	−	−	−	−	−	−	−
RVG4	−	−	−	−	−	−	−	−	−	−	−	−	−	−
UTMB1	−	−	−	−	−	−	−	−	−	−	−	+	−	−
UTMB2	−	−	−	−	−	−	−	−	−	−	−	−	−	−
UTMB4	+	−	−	−	−	−	−	−	−	−	−	−	−	−
UTMB7	−	−	−	−	−	−	−	−	−	−	−	−	−	−
LA^c^	−	−	−	−	−	−	−	−	−	−	−	−	−	−
EPEC^d^	+	−	−	−	nd	nd	nd	nd	nd	nd	nd	nd	nd	nd
*L. monocytogenes*	+	Nd	nd	nd	nd	nd	nd	nd	nd	nd	nd	nd	nd	nd
*S. aureus*	+	+	+	nd	nd	nd	nd	nd	nd	nd	nd	nd	nd	nd
*E. faecalis*	nd	+	nd	nd	+	+	+	+	+	+	+	+	+	nd
*P. acidilactici*	nd	Nd	nd	+	nd	nd	nd	nd	nd	nd	nd	nd	nd	nd
*E. faecium*	nd	Nd	nd	nd	Nd	nd	nd	nd	nd	nd	nd	nd	nd	+

+, positive amplification; −, negative amplification; nd, not determined.^c^LA, Lactobacillus acidophilus NCFM control strain.^d^EPEC, enteropathogenic *Escherichia coli*.

### 3.5. Functional characterization

The strains under study were evaluated for their antioxidant activity using the radical DPPH assay (Table 7). All the tested bacteria presented antioxidant activities ranging from 24 to 35%, which were superior in all the cases to that of the control strain *Lact. acidophilus* NCFM. In the galactosidase activity assay, all LAB strains, including the control *Lact. acidophilus* NCFM resulted in the development of blue colonies on the detection medium (Table 7), which was indicative of lactose hydrolysis. On the other hand, the control *Lact. acidophilus* NCFM presented a remarkable β-galactosidase activity comparable with the tested LAB strains. Additionally, in terms of cholesterol, the LAB strains resulted in the reduction of cholesterol with values ranging from 16.7 to 43.3% (Table 7). Among the tested LAB strains, *Lactiplantibacillus plantarum* RVG4 resulted in the highest reduction of cholesterol with 43.3%, a value which was statistically lower than that caused by the control strain *Lact. acidophilus* NCFM (48.5%).

**Table 7:** Functional traits of pulque lactic acid bacteria: antioxidant activity; β-Galactosidase activity; and cholesterol reduction.

Strain	Antioxidant activity (%)*	β-Galactosidase activity^a^	Cholesterol reduction (%)*
*Lact. paracasei* RVG1	26.1 ± 0.5 ^d^	+	16.7 ± 0.3 ^i^
*Lact. plantarum* RVG2	24.7 ± 0.6 ^e^	+	37.2 ± 0.3 ^d^
*Lact. plantarum* RVG4	26.3 ± 0.7 ^d^	+	43.3 ± 0.4 ^b^
*Lact. plantarum* UTMB1	28.6 ± 0.3 ^c^	+	36.2 ± 0.2 ^e^
*Lact. brevis* UTMB2	35.6 ± 0.6 ^a^	+	39.5 ± 0.3 ^c^
*Lact. paracasei* UTMB4	32.6 ± 0.2 ^b^	+	23.8 ± 0.4 ^h^
*Lact. paracasei* UTMB7	33.0 ± 0.6 ^b^	+	30.8 ± 0.3 ^g^
LA^b^	6.6 ± 0.2 ^f^	+	48.5 ± 0.0 ^a^

^*^ Results are expressed as the mean ± SD (*n* = 3). The letters correspond to the Tukey test results (*p* < 0.05), different letters in each column indicate significant difference in the Tukey test.

^a^ (−) no activity; (+) activity.

^b^ LA, *Lactobacillus acidophilus* NCFM control strain.

### 3.6. Multivariate analysis

#### 3.6.1. PCA and heatmap

**Figure 2:**
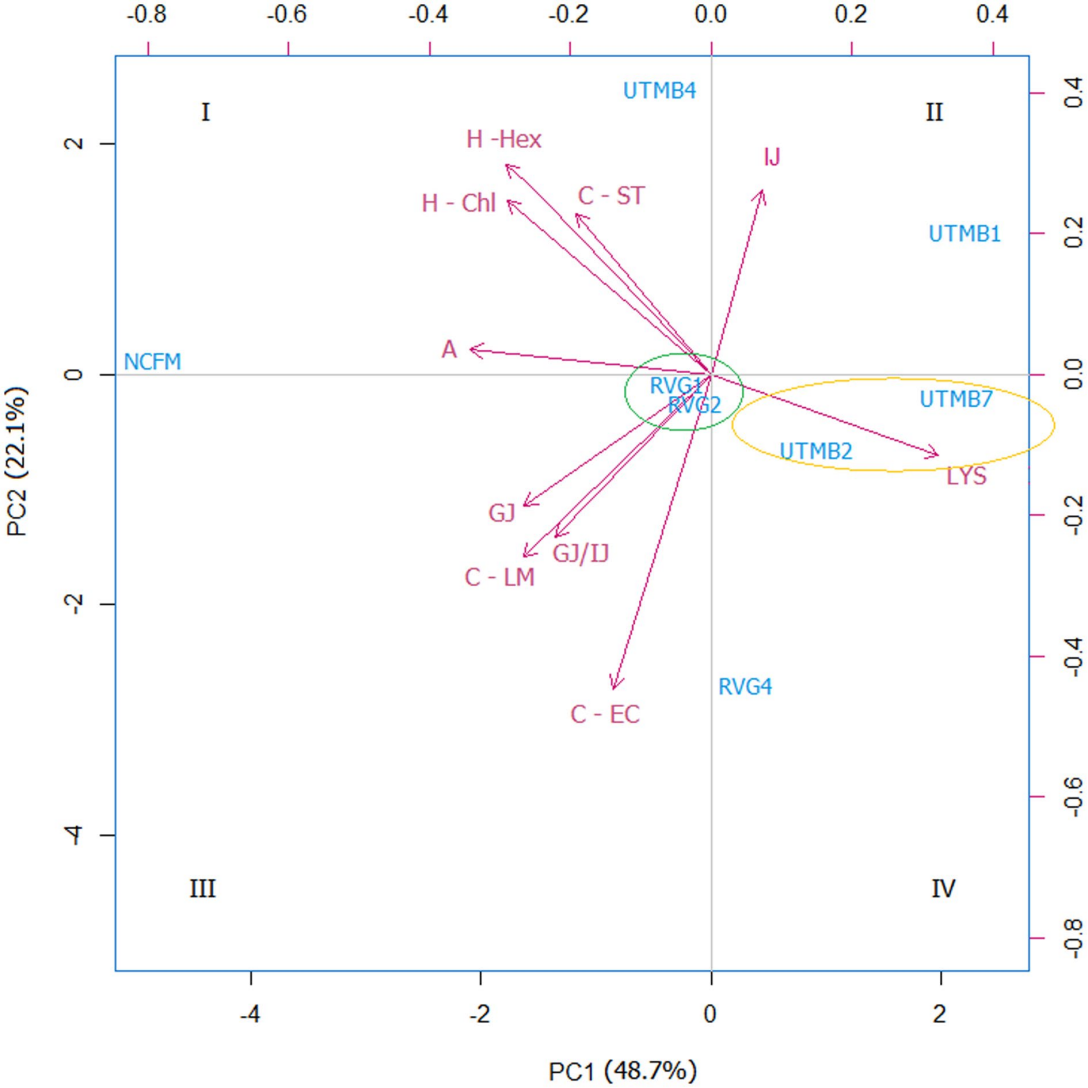
Principal component analysis (PCA) for the lactic acid bacteria from pulque and the positive control *Lactobacillus acidophilus* NCFM, considering the attributes for the determination of probiotic activity *in vitro*: resistance to gastric juice (GJ), intestinal juice (IJ),gastric juice/intestinal juice (GJ/IJ)lysozyme (LYS), as well as hydrophobicity (H; -Hex: hexane; -Chl: chloroform), auto-aggregation (A) and co-aggregation (C; −LM: *Listeria monocytogenes*; EC: EPEC; ST: *Salmonella Typhi*).

**Figure 3:**
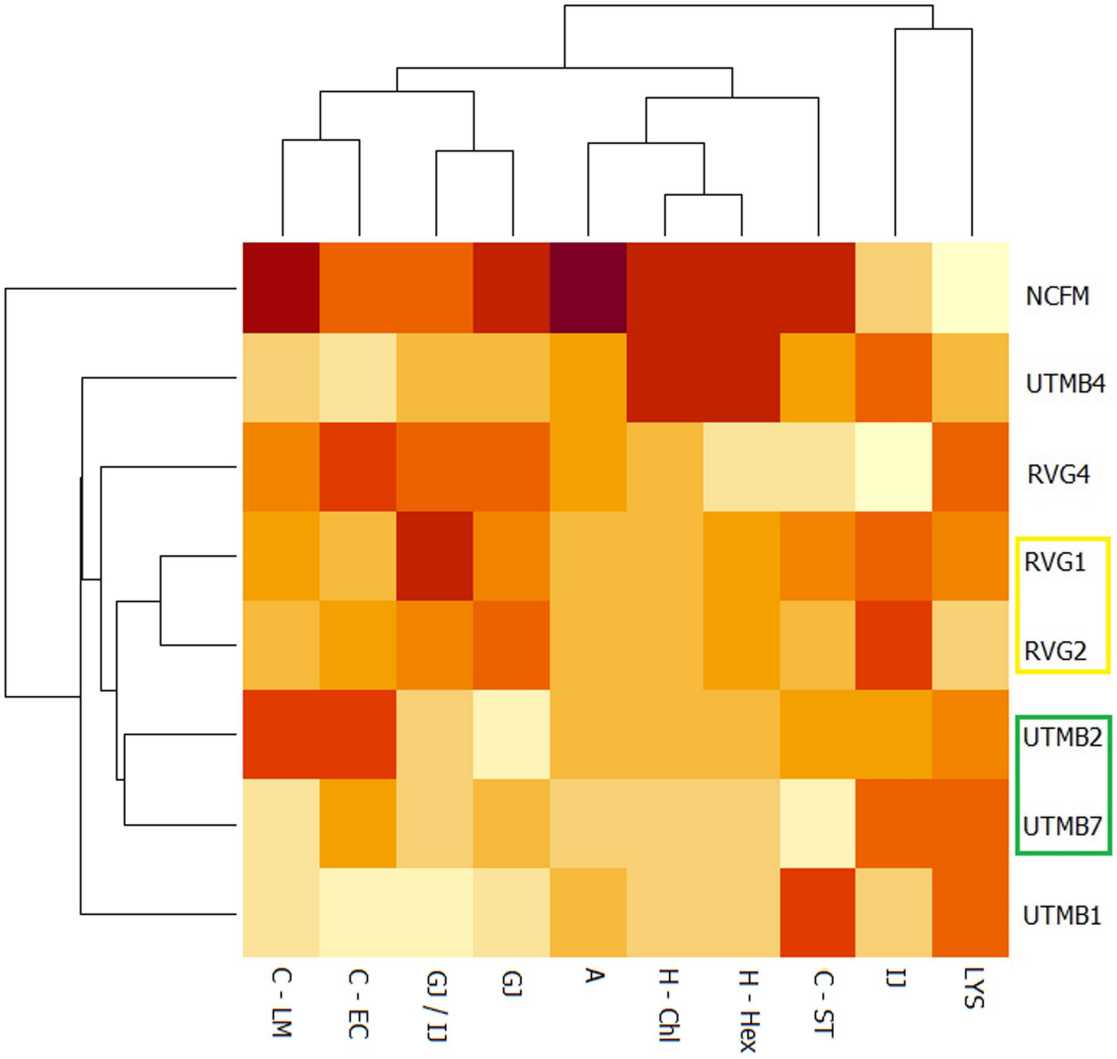
Heat map for the lactic acid bacteria from pulque and the positive control *Lactobacillus acidophilus* NCFM, considering the attributes for the determination of probiotic activity *in vitro*: resistance to gastric juice (GJ), intestinal juice (IJ),gastric juice/intestinal juice (GJ/IJ)lysozyme (LYS), as well as hydrophobicity (H; -Hex: hexane; -Chl: chloroform), auto-aggregation (A) and co-aggregation (C; −LM: *Listeria monocytogenes*; EC: EPEC; ST: *Salmonella Typhi*).

A principal component analysis (PCA) was performed on the data obtained from the probiotic characterization of the tested LAB strains and the control *Lact. acidophilus* NCFM. The parameters considered were the following: resistance to lysozyme, gastric juice, intestinal juice and gastric juice/intestinal juice, as well as hydrophobicity, auto-aggregation and co-aggregation. From the PCA analysis, two principal components (PC1 and PC2) were obtained, which covered a total variance of 70.8% (Figure 2). Component PC1 covers the maximum variance (48.7%) of the data, whereas component PC2. The projections of the seven LAB strains in the graph were grouped into four quadrants: I: NCFM and UTMB4; II: UTMB1; III: RVG1 and RVG2; IV: UTMB2, UTMB7 and RVG4. The probiotic characterization data were also represented in terms of color intensity variations using a heat map (Figure 3). The heat map provided the categorization of the LAB strains according to their probiotic attributes, in which the bacterial strains were grouped into 6 clusters: NCFM, UTMB4, RVG4, RVG1-RVG2, UTMB2-UTMB7 and UTMB1.

#### 3.6.2. Correlation analysis

**Figure 4:**
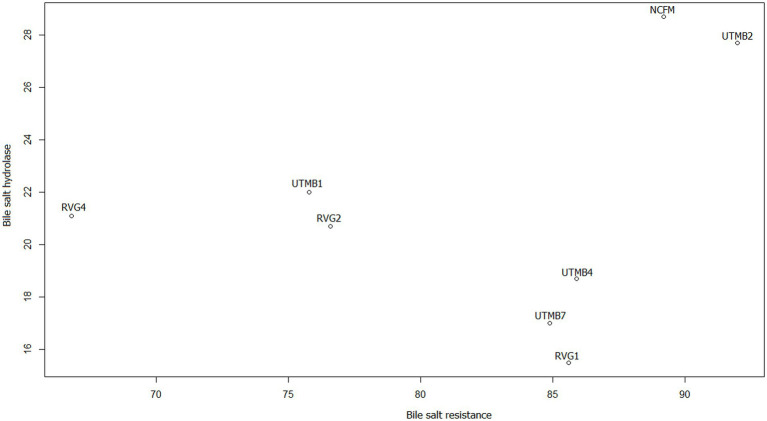
Scatter plot of the correlation between the variables bile salt resistance (%; BS 0.5%) (Ruíz-Ramírez et al., 2022) and bile salt hydrolase (mm of inhibition halo) enzyme production.

**Figure 5:**
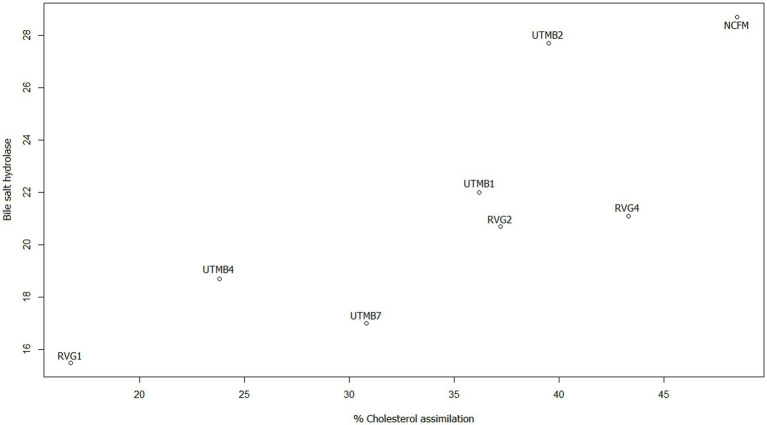
Scatter plot of the correlation between the variables bile salt hydrolase (mm of inhibition halo) enzyme production and cholesterol reduction (%).

In addition, scatter plots were made to obtain the correlation of the variables that according to the literature are related, this is the case of bile salt resistance, bile salt hydrolase activity and cholesterol reduction. Figure 4 shows the relationship between resistance to 0.5% bile salts (SB 0.5%) from our previous work (Ruíz-Ramírez et al., 2022) and the production of the enzyme bile salt hydrolase (BSH). The strains with the highest correlation coefficients for these variables are *Lact. acidophilus* NCFM, *Lact. brevis* UTMB2, *Lact. plantarum* RVG2 and *Lact. plantarum* UTMB1. The correlation between the variables BSH and cholesterol reduction was high for all the studied strains including the control (Figure 5).

## 4. Discussion

A number of species of lactic acid bacteria (LAB) belonging to the *Lactobacillaceae* family have been employed commercially as probiotic strains with diverse confirmed health benefits, which gives this bacterial group a great prominence in the food and pharmaceutical industries (Rezaei et al., 2020). In this work MALDI mass spectrometry was used for confirming the strain identification by the 16S rRNA genotyping. The identification results obtained by MS yielded quality rating values greater than two, which indicates high-confidence strain assignments.

Several studies carried out on the microbiota of pulque have reported a significant presence of *Lactobacillaceae* species reported in the present work (Escalante et al., 2016; Rocha-Arriaga et al., 2020). In a previous study (Ruíz-Ramírez et al., 2022), we reported that the strains isolated from pulque belonging to the species *Lact. plantarum, Lact. brevis* and *Lact. pasaracasei* presented diverse probiotic and antimicrobial activities *in vitro*. In this work, a series of *in vitro* tests were carried out on some of the LAB strains of pulque of our previous study to determine their probiotic potential in more detail as well as their functional and safety properties. The studied strains were subjected to the following stress conditions simulating the gastrointestinal tract: (i) the presence of lysozyme; (ii) gastric juice; (iii) intestinal juice, and (iv) the combined gastric and intestinal juice treatments.

The LAB strains tested in this work presented high survival rates in the presence of lysozyme, which in all cases were superior to that of the control strain *Lact. acidophilus* NCFM. Particularly, the strains *Lact. paracasei* RVG1 and *Lact. plantarum* RVG2 presented a 91% survival to lysozyme, a value that was considerably higher than those of the rest of the strains. Torres-Maravilla et al. (2016) evaluated the resistance to lysozyme (10 mg/L) of 14 LAB isolated from pulque reporting survival rates from 76 to 99.9%. In the study, the strains *Lact. plantarum* LBH1062 (99.7%) and *Lact. brevis* LBH1069 (99.9%) showed the highest survival percentages in the presence of lysozyme; results which are comparable with those of the present work.

The tested LAB strains presented survival rates to gastric juice conditions significantly lower (*p* < 0.05) from that of the commercial probiotic strain *Lact. acidophilus* NCFM (56.1%), with the *Lact. plantarum* RVG2 and *Lact. paracasei* UTMB7 strains presenting the highest survival rate (49%). Previously, Ruíz-Ramírez et al. (2022) evaluated these strains for their resistance to pH 2.0, obtaining survival percentages between 61 and 87%. Therefore, the results of this study indicate that in addition to the low pH, the proteolytic enzyme had a substantial contribution toward the inactivation of the LAB strains, a phenomenon that should be considered in the assessment of probiotic strain candidates. In this regard, bacterial microencapsulation can be used as a means to protect probiotic microorganisms from harsh environmental factors, such as gastric juice conditions (Vivek et al., 2023).

The tested LAB strains showed high resistance rates to intestinal juice, which were comparable with those reported in other studies carried out with LAB from other sources (Kumari et al., 2022; Zhang et al., 2022). The LAB strains *Lact. paracasei* RVG4 (99.4%), *Lact. brevis* UTMB2 (99.5%) and *Lact. paracasei* UTMB7 (99.9%) showed the highest resistance rates. In addition to this, a sequential test of the passage through the stomach and intestine (GJ/IJ) was performed: with this we could corroborate that the most stressful condition for LAB was the gastric juice, because most of the strains maintained the survival rate they had after passing through the stomach-simulated conditions. However, Zhang et al. (2022) also performed a sequential stomach and intestinal passage test and the authors reported decreased percent survival, indicating that their study strains have increased sensitivity to intestinal juice. These results confirm the importance of gastric juice in the survival of the tested microorganisms after passing through the simulated gastrointestinal tract.

One of the main mechanisms that is related to the resistance to bile salts by the LAB microorganisms is the production of bile salts hydrolase (BSH) enzymes (Ahn et al., 2003). The capacity of the tested LAB strains to produce BSH was evaluated using the agar diffusion method. While all the tested strains presented BSH lytic activity, the greatest activities were observed in the presence of the strains *Lact. plantarum* RVG2, *Lact. plantarum* RVG4, *Lact. plantarum* UTMB1 and *Lact. brevis* UTMB2, which comparable to that observed with the probiotic bacteria *Lact. acidophilus* NCFM. These results are in agreement with the work conducted by Vijayalakshmi et al. (2020) who evaluated the BSH activity of 9 LAB strains using a medium with 0.3% bile salts. The authors reported that the strains *Lact. rhamnosus, Lact. plantarum* and *Lact. casei* presented BSH activity.

The antibacterial activity of the tested LAB strains using *L. monocytogenes* LM-W207 as the target strain was comparable to other reports in the literature where LAB strains isolated from pulque were evaluated (Castro-Rodríguez et al., 2015; Giles-Gómez et al., 2016). In addition, in our previous study, these pulque strains showed antibacterial activity against *Salmonella enterica* serovar Typhi ATCC 9992 and EPEC 2348/69 (Ruíz-Ramírez et al., 2022). In the gastrointestinal tract, LAB bacteria will encounter a competitive niche for binding sites on the intestinal epithelium, so their antibacterial capacity provides an effective means of epithelial colonization (da Silva Ferrari et al., 2016). The antibacterial activity presented by the LAB strains is mainly explained by the capacity of these bacteria to produce diverse compounds, among which those of protein nature, such as bacteriocins and peptidoglycan hydrolases, have recently gained significant commercial and scientific interest (Ruíz-Ramírez et al., 2022).

The LAB strains of this study presented higher adhesion values compared to other studies performed on bacteria isolated from pulque (Torres-Maravilla et al., 2016). Of the studied strains, *Lact. brevis* UTMB2 showed the highest hydrophobicity value which was significantly lower (*p* < 0.05) than the commercial probiotic strain *Lact. acidophilus* NCFM for both solvents tested. Hydrophobicity is considered a criterion to select a probiotic strain because this attribute is related to the ability of LAB bacteria to auto-aggregate, which in turn is associated with the capacity of microorganisms to adhere to the intestinal epithelium (Kang et al., 2018; Vijayalakshmi et al., 2020).

In addition, the tested LAB strains presented low auto-aggregation percentages (17–34%), with the control strain *Lact. acidophilus* NCFM presenting remarkably high values (94%). Other authors (Collado et al., 2008; Śliżewska et al., 2021) have reported auto-aggregation rates for LAB strains between 30 and 50%, which are similar to the values of some of the LAB strains evaluated in the present study. Auto-aggregation has been associated with the ability of LAB strains to inhibit pathogenic microorganisms, which depends on the concentration of beneficial microorganisms in the mucosa of the gastrointestinal tract (Śliżewska et al., 2021).

The co-aggregation capacity of the LAB strains under study was evaluated using three test microorganisms, *S. enterica* serovar Typhi ATCC 9992, *L. monocytogenes* LM-W207 and EPEC 2348/69. Some of the studied LAB strains showed coaggregation values equal to those of the *Lact. acidophilus* NCFM control. The co-aggregation activity together with auto-aggregation constitutes a good indication of the adhesion capacity of LAB bacteria to epithelial cells (Zuo et al., 2015; Śliżewska et al., 2021).

Antibiotic resistance has increased considerably in modern societies and is now considered a global public health problem. This resistance has been attributed mainly to gene transfer mechanisms (Pradhan et al., 2020; Fan et al., 2023). Considering this, the LAB strains from pulque were evaluated against 15 antibiotics; a study in which all the strains displayed resistance to no more than 3 of the antibiotics tested. Most of the studied pulque LAB showed resistance to antibiotic quinolone group (ciprofloxacin and ofloxacin). Other authors showed a similar data with resistance to quinolones (Sharma et al., 2015; Duche et al., 2023). Sharma et al. (2015) evaluated antibiotic resistance of *Lactobacillus* sp. strains from commercial probiotic products, and reported high resistance to quinolones, mostly to nalidixic acid. In general the mechanisms of bacterial resistance to quinolone include: (i) point mutations in the quinolone resistance-determining regions (QRDRs) of the genes encoding gyrase (*gyrA* and *gyrB*) and topoisomerase (*parC* and *parE*); (ii) bacterial overexpression of efflux pump systems that reduces internal accumulation; and (iii) plasmid-mediated quinolone resistance (Hernández et al., 2011). But the LAB quinolone resistance is closely related to point mutations in the QRDRs and to the overexpression of efflux pump systems (Jiang et al., 2016). Therefore, quinolone resistance is mainly associated with not transferable intrinsic resistance mechanisms, this feature may be considered an advantage for a probiotic bacterium (Duche et al., 2023). These results are favorable compared with those reported by Colombo et al. (2020) who evaluated the resistance of various bacteria of the *Lactobacillaceae* family to 12 antibiotics, reporting that the LAB strains were not resistant to more than 7 antibiotics.

Most of the LAB strains from pulque presented ɣ-hemolytic activity, except for *Lact. plantarum* UTMB1 and *Lact. plantarum* RVG2, which presented α-hemolytic activity. On the other hand, as expected, the positive control *L. monocytogenes* presented β-hemolytic activity. Therefore, from these results, it can be inferred that most of the tested LAB strains can be considered safe in terms of their hemolytic activity. In addition, certain types of hemolytic activity (β-hemolytic and ɣ-hemolytic) are considered relevant virulence factors that are mainly present in pathogenic microorganisms (Touret et al., 2018).

Additionally, to further corroborate the safety of the LAB strains, the cells´ expression of genes related to antibiotic resistance and virulence factors were determined by PCR. Among the studied bacteria, only the strain *Lact. paracasei* UTMB4 showed amplification of one antibiotic resistance gene (*mecA*). Resistance to beta-lactams is associated with the *mecA, blaZ, bla* and *int-Tn (Tn916/Tn1545)* genes (Duche et al., 2023), but in this work only the *mecA* gene was evaluated. Thus further studies are necessary to obtain conclusive results regarding the resistance of the strains to beta-lactams. On the other hand, only *Lact. plantarum* strain UTMB1 amplified the gene *agg* associated with virulence factors, which encode for the synthesis of adhesin, a surface protein. This gene can only be induced by pheromones involved in the binding of the donor cell to plasmid-free acceptor cells, thus promoting cell aggregation (Waters and Dunny, 2001); however, genes associated with pheromone production were not expressed in the strain UTMB1.

For a comprehensive assessment of the beneficial capacity of a LAB strain, besides the probiotic activity and safety characterization, it would be highly desirable that the strain provides an additional beneficial contribution toward human health, i.e., a functional activity (Kaur et al., 2017). The antioxidant activities of the LAB strains under study were superior to that of the commercial probiotic strain *Lact. acidophilus* NCFM, with values greater than 20%, which agrees with similar reports in the literature for LAB strains (Kaur et al., 2017; Rezaei et al., 2020). Antioxidant activity by probiotics is associated with the regulation of gastrointestinal tract conditions, thus acting as a protective barrier against pathogenic microorganisms (Kaur et al., 2017).

Lactose digestion in humans occurs by the hydrolytic action of the enzyme β-galactosidase, so the deficiency of the latter is associated with lactose intolerance, which causes gastric discomfort such as abdominal pain, diarrhea, flatulence, nausea, etc., in the sufferer (Gheytanchi et al., 2010; Kaur et al., 2017). Considering that all the pulque LAB strains and the positive control presented β-galactosidase activity, the consumption of these microorganisms can assist in alleviating lactose intolerance (Kaur et al., 2017).

Hypercholesterolemia is one of the main causes of cardiovascular diseases worldwide. According to the relevant literature, a 1% decrease in cholesterol could reduce the risk of coronary heart disease by 2 to 3% (Sakandar et al., 2019). In recent studies, it has been reported that some LAB strains can reduce the level of cholesterol in the intestine. Among the pulque strains, *Lactiplantibacillus plantarum* RVG4 resulted in the highest reduction rate (43.3%), which was significantly higher (*p* < 0.05) than the rest of the strains studied, but moderately lower than the commercial probiotic strain *Lact. acidophilus* NCFM (48.5%). Other authors have reported cholesterol reduction percentages between 50 and 59% by *Lact. casei* marine strains (Das et al., 2016). Li et al. conducted *in vitro* and *in vivo* studies of the probiotic properties of LAB strains isolated from traditional Chinese sourdough. The authors performed a cholesterol reduction assay and reported values of 20–30% for *Lact. plantarum* strains, which were similar to those obtained in the present study.

For a systematic selection of the best probiotic candidates, a multivariate analysis was performed using PCA and heat mapping assessment. The strains were grouped into four quadrants considering their performance in the probiotic activity tests. The attributes hydrophobicity, auto-aggregation, gastric juice and gastric juice/intestinal juice were determinant for the categorization of the LAB strains since these parameters accounted for the greatest variability of the tested bacteria. According to the PCA analysis (Figure 2), the strains *Lact. acidophilus* NCFM and *Lact. paracasei* UTMB4 are located in quadrant I, presenting the best results in terms of cell-surface characteristics, and in general, favorable results in the rest of the probiotic activity attributes. Besides this, considering the PCA and heat map analyses, the strain RVG4 is the closest bacteria to the strain UTMB4; therefore, the former strain can be considered as the second-best probiotic candidate from the pulque strains tested in this study. Additionally, the strains RVG1 and RVG2 constitute the third closest group to the commercial probiotic strain *Lact. acidophilus* NCFM. Moreover, taking into account the positioning of this cluster in the PCA plot as well as its color intensity pattern presented in the heat map, this cluster presents intermediate values in most of the probiotic activity attributes. Accordingly, the strains RVG1 and RVG2 can be considered as good probiotic candidates. A very decisive factor for the final positioning of the lactic acid strains was their resistance to gastric juice; however, for improving bacterial resistance to this stressing condition, cell microencapsulation can be employed (Vivek et al., 2023). Taking this into account, the strain UTMB2 (quadrant IV) can be also considered as a good probiotic candidate, especially due to its remarkable cell surface properties.

Finally, the variables of bile salt resistance, BSH synthesis and cholesterol reduction were analyzed. According to the literature, bile salt resistance and cholesterol reduction are related to BSH production. As mentioned above, the main mechanism for bile salt resistance has been reported to be the synthesis of bile salt hydrolase (BSH) enzymes. In addition, a proposed mechanism for cholesterol reduction is the enzymatic deconjugation of bile acids by BSH enzymes (Ahn et al., 2003; Abushelaibi et al., 2017). Therefore, in this study scatter plots of these variables indicated that the correlation between BSH and cholesterol reduction was greater (Figure 5) than that between bile salt resistance and BSH (Figure 4). It can be observed that the pulque strain *Lact. brevis* UTMB2 presented both the highest resistance to bile salts and one of the largest production of the BSH enzyme, with values which were similar to those of the probiotic control strain. In addition, BSH production was positively correlated to cholesterol reduction in all strains studied, including the commercial probiotic strain. On the other hand, the strain with the highest BSH production and the highest percentage of cholesterol reduction was the NCFM strain, which was closely followed by the pulque strain UTMB2. In conclusion, the synthesis of BSH enzymes by LAB bacteria is the main mechanism involved in improving the cell’s resistance to bile salts and cholesterol reduction.

This study provides evidence of the probiotic potential of LAB of the Lactobacillaceae family isolated from pulque and thus demonstrated that pulque can be considered as an excellent source of beneficial microorganisms. The tested LAB strains displayed favorable results in all the probiotic activity and safety tests performed in this study, as compared with the commercial probiotic strain *Lact. acidophilus* NCFM and other isolates from pulque previously reported in the literature. In general, considering the clustering results obtained with both multivariate analysis tools utilized in this work (PCA and heat mapping) the pulque strains with the highest probiotic potential were *Lacticaseibacillus paracasei* UTMB4, *Lactiplantibacillus plantarum* RVG4 and *Levilactobacillus brevis* UTMB2; however, the UTMB4 strain expressed a gene related to resistance to beta-lactam antibiotics. Considering the above, the tested LAB strains from pulque may be employed in the elaboration of diverse fermented and functional foods to provide them with specific probiotic characteristics. However, studies are needed *in vivo* to be able to assert the probiotic nature and safety characteristics of the tested strains. Overall, this research contributes to the scientific valorization of the probiotic potential of pulque which may help to the commercial resurgence of this ancestral fermented beverage.

## Data availability statement

The datasets presented in this study are deposited in the NCBI GenBank repository. Accession numbers are available in Supplementary Table S2.

## Author contributions

YR-R, EP-A, and RV-B: experimental design and development. YR-R and CC-G: statistical analysis, EP-A, RV-B, and MC: project supervision. YR-R, EP-A, CC-G, MC, and RV-B: manuscript drafting, reviewing, and editing. All authors contributed to the article and approved the submitted version.

## Funding

YR-R thanks Consejo Nacional de Ciencia y Tecnología (CONACyT) Mexico for the support of her doctoral studies through the scholarship No. 714207/ 754129. MC was supported in part by the Ministry of Science and Higher Education of the Russian Federation (Project Number 075-15-2019-1880).

## Conflict of interest

The authors declare that the research was conducted in the absence of any commercial or financial relationships that could be construed as a potential conflict of interest.

## Publisher’s note

All claims expressed in this article are solely those of the authors and do not necessarily represent those of their affiliated organizations, or those of the publisher, the editors and the reviewers. Any product that may be evaluated in this article, or claim that may be made by its manufacturer, is not guaranteed or endorsed by the publisher.
