# Supplementary material for: Probiotic and functional potential of lactic acid bacteria isolated from pulque and evaluation of their safety for food applications
Source: Front Microbiol. 2023 Sep 12;14:1241581. doi: 10.3389/fmicb.2023.1241581 (PMC10536145; doi:10.3389/fmicb.2023.1241581)
Supplement: Supplementary file 1 [file Table_1.pdf]

# Probiotic and functional potential of lactic acid bacteria isolated from pulque and evaluation of their safety for food applications

**Yesica Ruiz-Ramírez[1], Rogelio Valadez-Blanco[2], Concepción Calderón-García[2], Michael Leonidas Chikindas[3,4,5], Edith Ponce-Alquicira[1,*]**

[1]Departamento de Biotecnología, Universidad Autónoma Metropolitana, Unidad Iztapalapa, San Rafael Atlixco No.186, Col. Vicentina, 09340, Iztapalapa, Ciudad de México, México

[2]Instituto de Agroindustrias, Universidad Tecnológica de la Mixteca, Carretera a Acatlima km 2.5, 69000 Huajuapan de León, Oaxaca, México

[3]Health Promoting Naturals Laboratory, School of Environmental and Biological Sciences, Rutgers State University, 65 Dudley Road, New Brunswick, NJ 08901, USA

[4]Center for Agrobiotechnology, Don State Technical University, Gagarin Square 1, Rostov-on-Don 344002, Russia

[5]Department of General Hygiene, I.M. Sechenov First Moscow Medical University, Bolshaya Pirogovskaya Str., 19/1, Moscow 119146, Russia

***Correspondence:** Edith Ponce-Alquicira pae@xanum.uam.mx

**Supplementary material 1** Auto-aggregation capacity (%) at 2, 4, 6, 20 y 24 h of lactic acid bacteria from pulque and the control *Lactobacillus acidophilus* NCFM.

| **Strain** | **Time (h)** | | | | |
|---|---|---|---|---|---|
| | **2** | **4** | **6** | **20** | **24** |
| *Lact. paracasei* RVG1 | 2.6 ± 0.6 | 15.3 ± 0.1 | 16.2 ± 0.1 | 16.8 ± 0.3 | 17.0 ± 0.3 |
| *Lact. plantarum* RVG2 | 4.3 ± 0.0 | 14.4 ± 0.3 | 11.7 ± 0.0 | 25.9 ± 0.1 | 27.0 ± 0.1 |
| *Lact. plantarum* RVG4 | 3.4 ± 0.0 | 6.8 ± 0.1 | 8.2 ± 0.1 | 17.0 ± 0.2 | 21.9 ± 0.2 |
| *Lact. plantarum* UTMB1 | 2.6 ± 0.4 | 3.1 ± 0.1 | 4.1 ± 0.0 | 11.7 ± 0.1 | 15.8 ± 0.3 |
| *Lact. brevis* UTMB2 | 3.9 ± 1.0 | 14.5 ± 0.8 | 30.9 ± 0.2 | 32.0 ± 0.2 | 34.3 ± 0.5 |
| *Lact. paracasei* UTMB4 | 3.6 ± 1.0 | 10.5 ± 0.1 | 12.8 ± 0.0 | 15.9 ± 0.1 | 20.3 ± 0.1 |
| *Lact. paracasei* UTMB7 | 2.1 ± 0.4 | 9.7 ± 0.1 | 16.6 ± 0.1 | 18.6 ± 0.1 | 19.5 ± 0.0 |
| NCFM | 1.7 ± 0.1 | 27.1 ± 0.0 | 39.2 ± 0.1 | 87.6 ± 0.3 | 94.1 ± 0.1 |
