# Supplementary material for: Probiotic and functional potential of lactic acid bacteria isolated from pulque and evaluation of their safety for food applications
Source: Front Microbiol. 2023 Sep 12;14:1241581. doi: 10.3389/fmicb.2023.1241581 (PMC10536145; doi:10.3389/fmicb.2023.1241581)
Supplement: Supplementary file 2 [file Table_2.pdf]

Probiotic and functional potential of lactic acid bacteria isolated from pulque and evaluation of their safety for food applications

Yesica Ruiz-Ramírez[1], Rogelio Valadez-Blanco[2], Concepción Calderón-García[2], Michael Leonidas Chikindas[3,4,5], Edith Ponce-Alquicira[1,*]

[1]Departamento de Biotecnología, Universidad Autónoma Metropolitana, Unidad Iztapalapa, San Rafael Atlixco No.186, Col. Vicentina, 09340, Iztapalapa, Ciudad de México, México

[2]Instituto de Agroindustrias, Universidad Tecnológica de la Mixteca, Carretera a Acatlima km 2.5, 69000 Huajuapan de León, Oaxaca, México

[3]Health Promoting Naturals Laboratory, School of Environmental and Biological Sciences, Rutgers State University, 65 Dudley Road, New Brunswick, NJ 08901, USA

[4]Center for Agrobiotechnology, Don State Technical University, Gagarin Square 1, Rostov-on-Don 344002, Russia

[5]Department of General Hygiene, I.M. Sechenov First Moscow Medical University, Bolshaya Pirogovskaya Str., 19/1, Moscow 119146, Russia

***Correspondence:** Edith Ponce-Alquicira pae@xanum.uam.mx

Supplementary material 2 Co-aggregation capacity (%) with EPEC, *Salmonella* and *Listeria innocua* at 2, 4, 6, 20 y 24 h of lactic acid bacteria from pulque and the control *Lactobacillus acidophilus* NCFM.

***Escherichia coli* (EPEC) 2348/69**

**Strain**	**Time (h)**				
	**2**	**4**	**6**	**20**	**24**
Lact. paracasei RVG1	38.4 ± 0.4	41.7 ± 0.1	44.7 ± 0.0	54.9 ± 0.1	58.6 ± 0.1
Lact. plantarum RVG2	39.4 ± 0.3	45.1 ± 0.1	48.1 ± 0.1	63.8 ± 0.1	64.9 ± 0.1
Lact. plantarum RVG4	41.8 ± 0.4	45.9 ± 0.1	49.5 ± 0.0	59.5 ± 0.1	61.4 ± 0.1
Lact. plantarum UTMB1	41.3 ± 0.1	44.1 ± 0.1	46.8 ± 0.1	61.3 ± 0.1	62.3 ± 0.1
Lact. brevis UTMB2	37.9 ± 0.2	40.3 ± 0.1	45.3 ± 0.1	58.1 ± 0.1	59.5 ± 0.1
Lact. paracasei UTMB4	40.6 ± 0.4	42.3 ± 0.1	45.5 ± 0.1	63.0 ± 0.1	64.4 ± 0.1
Lact. paracasei UTMB7	40.9 ± 0.4	44.7 ± 0.1	49.2 ± 0.0	60.3 ± 0.0	62.2 ± 0.1
NCFM	43.3 ± 0.3	48.6 ± 0.1	54.1 ± 0.1	63.7 ± 0.1	64.1 ± 0.0

***Listeria monocytogenes* LM-W207**

**Strain**	**Time (h)**				
	**2**	**4**	**6**	**20**	**24**
Lact. paracasei RVG1	61.6 ± 0.1	66.3 ± 0.3	65.9 ± 0.1	82.3 ± 1.2	83.7 ± 0.5
Lact. plantarum RVG2	61.9 ± 0.1	68.9 ± 0.0	69.8 ± 0.1	85.8 ± 0.0	85.8 ± 0.2
Lact. plantarum RVG4	59.6 ± 0.8	66.3 ± 0.8	67.7 ± 0.3	85.6 ± 0.8	85.3 ± 0.3
Lact. plantarum UTMB1	60.0 ± 0.6	63.3 ± 0.0	65.3 ± 0.8	81.6 ± 0.1	83.6 ± 0.1
Lact. brevis UTMB2	59.9 ± 0.1	64.0 ± 0.2	65.3 ± 0.5	83.7 ± 0.4	83.8 ± 0.3
Lact. paracasei UTMB4	61.4 ± 0.4	64.7 ± 0.4	66.4 ± 0.3	85.6 ± 0.5	86.5 ± 0.0
Lact. paracasei UTMB7	59.3 ± 0.6	66.7 ± 0.6	68.8 ± 0.3	84.8 ± 0.6	84.7 ± 0.0
NCFM	61.7 ± 0.5	70.2 ± 0.4	72.4 ± 0.3	87.2 ± 0.3	87.6 ± 0.4

***Salmonella enterica* serovar Typhi ATCC 9992**

**Strain**	**Time (h)**				
	**2**	**4**	**6**	**20**	**24**
Lact. paracasei RVG1	60. ± 0.5	65.2 ± 0.1	66.8 ± 0.5	85.9 ± 1.1	87.3 ± 0.4
Lact. plantarum RVG2	64.7 ± 0.4	66.6 ± 0.5	76.2 ± 0.3	83.4 ± 0.3	83.6 ± 0.2
Lact. plantarum RVG4	59.9 ± 0.4	64.2 ± 0.6	73.2 ± 0.3	85.5 ± 0.1	86.5 ± 0.2
Lact. plantarum UTMB1	62.3 ± 0.1	62.8 ± 0.0	64.5 ± 0.5	83.1 ± 0.1	83.4 ± 0.1
Lact. brevis UTMB2	58.8 ± 0.9	63.2 ± 0.1	71.3 ± 0.2	85.1 ± 0.1	85.4 ± 0.1
Lact. paracasei UTMB4	63.0 ± 0.3	66.8 ± 0.1	70.8 ± 0.8	84.2 ± 0.1	85.9 ± 0.2
Lact. paracasei UTMB7	59.5 ± 0.6	63.5 ± 0.4	66.4 ± 1.3	83.7 ± 0.7	84.7 ± 0.3
NCFM	62.7 ± 1.1	70.2 ± 0.1	75.1 ± 0.9	87.2 ± 0.2	88.2 ± 0.4
